# Enhancing Access to Family Planning Services in Uganda Through Community Health Extension Workers: Protocol for a Pilot Evaluation

**DOI:** 10.2196/81627

**Published:** 2025-12-04

**Authors:** Lydia Kabwijamu, Steven Ndugwa Kabwama, Fredrick Makumbi, Roselline Achola, Andrew Tusubira, Sarah Nabukeera, Christine K Nalwadda

**Affiliations:** 1 Department of Community Health & Behavioral Sciences, Makerere University School of Public Health, Kampala, Uganda; 2 Department of Global Public Health, Karolinska Institutet, Tomtebodavägen 18A, Solna, Stockholm, 17177, Sweden, 46 707578093; 3 Department of Epidemiology and Biostatistics, Makerere University School of Public Health, Kampala, Uganda; 4 Ministry of Health, Kampala, Uganda

**Keywords:** community health workers, contraceptives, family planning, Uganda, evaluation, protocol, reproductive health

## Abstract

**Background:**

In Uganda, 22% of all women of reproductive age have an unmet need for family planning services. Access to contraceptive services, especially long-term reversible contraceptives such as implants, remains a challenge. The number of trained health providers is also not sufficient to address the needs for contraception. The Uganda Ministry of Health implemented a community-based implant provision pilot project where community health extension workers (CHEWs) were trained and accredited to insert implants at community level.

**Objective:**

This study aims to evaluate the implementation and acceptability of stakeholders toward task shifting the provision of family planning implants to CHEWs in Uganda.

**Methods:**

The evaluation will use a cross-sectional design using both quantitative and qualitative methods. The quantitative component will use a noninferiority design, whereas the qualitative component will use a descriptive approach. The noninferiority design involves a comparison of the competence of the currently authorized cadre to offer the service to the proposed cadre (CHEWs). Compared with a randomized controlled trial, the noninferiority design is more appropriate for this evaluation because the CHEWs and the authorized cadre are not comparable in terms of level of training and competencies. The authorized cadre has gone through formal training, which is not comparable with the training the CHEWs have received, and so the comparison is such that the competencies of the CHEWs are noninferior or at most equal to the competencies of the authorized cadre. Quantitative data will be collected among 92 CHEWs and 92 qualified health workers using performance assessment checklists and practice-based questionnaires that were developed based on the training manuals. Competency will be measured on a continuous scale and summarized as mean (SD) scores. Qualitative data will be collected through key informant interviews (n=23), in-depth interviews (n=24), and focus group discussions (n=18). Qualitative data will be analyzed using thematic analysis following the framework method for the analysis of qualitative data using ATLAS.ti (version 9).

**Results:**

Preliminary findings indicate improved confidence and capacity of community health workers to provide implants despite challenges such as poor waste disposal, record keeping, and data management. By August 2025, training of research assistants had been concluded, and data collection had started. We anticipate that the data collection will be completed by the end of October 2025, the data analysis will be completed by November 2025, and the final results will be published by December 2026.

**Conclusions:**

This pilot will generate contextual information that can be used to improve access to family planning services at the community level.

## Introduction

### Background

The Sustainable Development Goals target 3.7 aimed to achieve 75% of the global demand for contraception by 2030 [1,2]. However, many women of reproductive age might never get access to quality contraceptive services and commodities for reasons such as insufficient information about available methods, stigma [3], shortage of family planning commodities, and few adequately trained health providers. A modeling study estimated that the global prevalence of modern contraceptive use among 1.9 billion women of reproductive age increased from approximately 55.0% in 2000 to 57.1% in 2019 [4]. However, the study also noted that approximately 164 million women of reproductive age still face unmet need for family planning and that Sub-Saharan Africa experiences a disproportionately higher proportion of women with an unmet need for family planning (23.1% [5]) compared with other regions such as Asia and Latin America, where the unmet need is approximately 10% [4].

In many low- and middle-income countries, the number of trained health providers is not sufficient to address the need for contraception [6,7]. In addition, less than half of the available trained health workers serve rural areas, where a significant proportion of the population resides [7,8]. To address this challenge, the World Health Organization acknowledges task shifting as an innovative approach to promote and accelerate equitable access to care, especially in low- and middle-income countries. It is defined as “the rational redistribution of tasks among health workforce teams,” where tasks are moved, transferred, or delegated, as appropriate, from trained and qualified health workers to other health workers with shorter training and fewer qualifications [8], to make more efficient use of the available human resources for health and improve service access.

In 2010, Uganda developed ambitious goals and targets for the transformation of its people and the achievement of a modern and prosperous society as per Vision 2040. Between 2010 and 2040, the country aimed to reduce the maternal mortality rate from 336 to 15 deaths per 100,000 live births, increase modern contraceptive prevalence rates from 35% in 2016 to 50% in 2030, and reduce unmet need for family planning from 28% in 2016 to 10% by 2030 [9]. To achieve these targets, various strategies have been instituted, including scaling up self-care and task shifting through engaging community health extension workers (CHEWs) to support service access and increase family planning uptake at the community level.

### Community-Based Implant Provision Project

The Uganda Ministry of Health developed a CHEWs strategy to support the achievement of the ambitious Vision 2040 and the 2030 Sustainable Development Goals targets. The objective of the strategy is to competently train and launch a cadre of health care providers, known as CHEWs, to deliver quality, preventive, promotive, and selected basic curative health services at the community level, including providing contraceptives.

The Uganda Ministry of Health implemented a pilot community-based implant project, which involved training CHEWs to provide long-acting reversible contraceptives. Specifically, CHEWs were trained to insert family planning implants and refer clients who needed services of removing the implants to health facilities. This approach has previously been tested in Ethiopia [10] and Nigeria [11]. However, there is a need to generate context-relevant evidence in Uganda to support the community-level provision of implants, even as global health stakeholders advance task shifting and sharing [12,13], community-based service delivery, and self-care for sexual and reproductive health.

The community-based implant provision project was piloted in Lira City, Kyotera, and Namutumba districts in Uganda. All CHEWs in the aforementioned districts underwent additional training specific to family planning focused on implant insertion. The additional training focused on implant provision and was designed to equip the CHEWs with knowledge, skills, and clinical competencies to provide Implanon NXT (Organon Pharma Limited) insertion as well as appropriate referral for implant removal. The training on implant insertion was provided for 8 days, with 4 days of classroom-based learning and 4 days of facility-based practicum. The training was delivered by national-level trainers using a training manual developed by the Uganda Ministry of Health. After successful training, the CHEWs were accredited and deployed at their immediately supervising health facility to conduct implant insertion services. By the end of the training, the CHEWs were expected to have acquired knowledge and demonstrate the following clinical competencies: (1) provide effective client counseling sessions addressing concerns and misconceptions, for informed choice and obtain consent from at least 3 to 5 women, (2) perform comprehensive client screening and medical eligibility for Implanon NXT, (3) prepare the insertion site, (4) perform Implanon NXT insertion in 5 clients using correct techniques, (5) provide clear postinsertion care instructions to 5 clients, (6) manage clients with family planning–related side effects and provide appropriate referral pathways (optional), (7) accurately and appropriately record the family planning services provided in family planning registers and client cards (5 records), and (8) maintain a safe and sterile environment during 5 procedures.

The project was facility-based, whereby CHEWs were stationed at their supervising facilities within their communities. During the duration of the intervention, the CHEWs worked alongside and were supervised by health workers to offer Implant NXT contraception to any woman who required it, was eligible, and consented. The CHEWs were then accredited to offer the services independently.

### General Objective

The overall objective is to evaluate the implementation of a community-based implant intervention to inform efforts aimed at task shifting the provision of long-term reversible family planning methods in Uganda.

### Specific Objectives

The specific objectives of the study are to (1) explore the perceptions of the stakeholders toward implant provision task shifting to the CHEWs (stakeholders including the CHEWs, clients, health workers or professionals, and policymakers), (2) assess the acceptability of the community-based implant insertion by CHEWs among the stakeholders (health workers at the facility and family planning clients in the community), and (3) determine the competence of the CHEWs regarding insertion of implants following training.

## Methods

### Study Site

**Figure 1.**
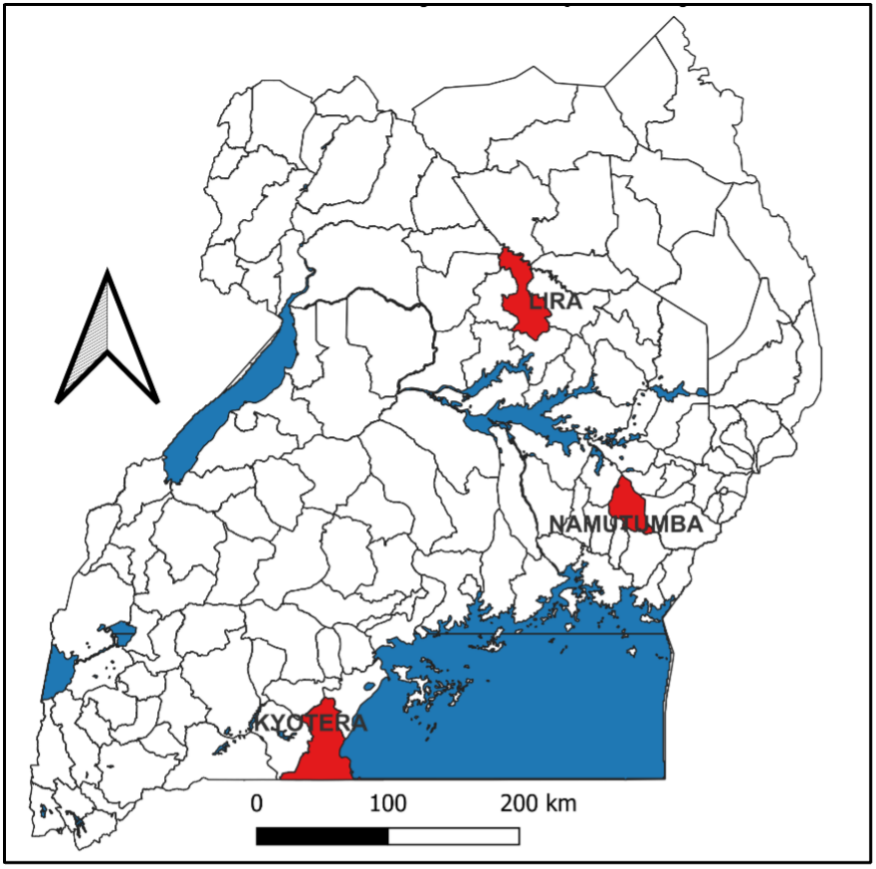
A map of Uganda showing the districts included in the community-based implant project.

The evaluation will be conducted in 3 pilot districts in the regions of East (Namutumba), North (Lira Municipality), and South (Kyotera) (Figure 1). These districts were selected because training and certification of the CHEWs have been completed and the electronic Community Health Information System has been rolled out.

### Study Design

The evaluation will use a convergent parallel mixed-method cross-sectional design using both quantitative and qualitative methods. The quantitative component will use a noninferiority design [14]. This involves a comparison of the competence of the currently authorized cadre to offer the service to the proposed cadre (CHEWs). The noninferior design is methodologically more rigorous than pre-post designs that have been used for assessments in previous studies [10,11].

The qualitative component will use the descriptive qualitative study approach. The descriptive qualitative study approach is an emerging approach that has been used in health care research to understand or identify participants’ experiences and perceptions [15] of the intervention or why the intervention worked or did not work and how the intervention might be improved [16].

### Study Population

The study population will include trained CHEWs, clients, health workers, program managers, and policymakers.

### Inclusion Criteria

The evaluation will include CHEWs who were trained and engaged in implant insertion as well as health workers who were engaged in the intervention as either trainers or supervisors of the CHEWs. Health workers engaged in the provision of long-term reversible family planning or those at the policy level at the national and district levels will also be included. The evaluation will also include clients defined as women of reproductive age who have obtained family planning services provided by CHEWs. To obtain perspectives from spouses of family planning users, men who are aged ≥18 years and in a relationship or union with a woman will also be included in the evaluation.

### Exclusion Criteria

Critically ill clients or those deemed unable to answer questions due to living with cognitive disabilities will be excluded. All potential respondents who refuse to respond or do not consent, or those who will not be available during the duration of the study, will be excluded.

### Sample Size Estimation

#### Quantitative Data

##### Sample Size Estimation: Noninferiority Design

CHEWs will be assessed on their competency to insert implants, under some domains with corresponding items, following training in the selected districts. The assessment of the competency of the CHEWs will be compared to the competency of the qualified health facility providers in the cadre currently authorized to insert implants. Competency will be measured on a continuous scale and summarized as mean (SD) scores. The competency of CHEWs should not be *worse than* that of the authorized cadre, within a margin of acceptable difference (-*d*_*NI*_), accounting for the mean (SD) score competency of authorized cadres (*μ*_*B*_) and that of CHEWs (*μ*_*A*_), and a 1-sided test.

We assumed an SD (*σ*^2^) of the competency scores, and the noninferiority limit (-*d*_*NI*_) was set at −10. At a power of 90% and 1-sided 95% CI, this resulted in a total of 138 participants (69, 50%, cadres authorized to provide implants and 69, 50%, CHEWs) needed to demonstrate that there is truly no difference between the competency of the cadres authorized and the trained CHEWs. We adjusted for a nonresponse rate of 10% and a design effect of 1.2 because multiple CHEWs selected from similar settings and cadres authorized selected from the same health facilities observations will be correlated. This adjustment will result in a minimum of (138×1.2)/0.9=184 (92 CHEWs and 92 authorized cadres).

Compared with a randomized trial, the noninferiority design is more appropriate for this evaluation because it would be a false assumption to randomize participants to receive the service by the CHEWs or the authorized cadre because the 2 groups are not comparable in terms of level of training and competencies. The authorized cadre has gone through formal training, which is not comparable with the training the CHEWs have received; therefore, the best approach for any comparison is such that the competencies of the CHEWs are noninferior or at most equal to the competencies of the authorized cadre.

##### Qualitative Sample

The qualitative approach will be used to obtain data on perceptions and acceptability of the intervention among stakeholders. Qualitative data will be collected using focus group discussions (FGDs), in-depth interviews (IDIs), and key informants. A minimum of 6 FGDs will be conducted among men in relationships and among women of reproductive age, giving a cumulative total of at least 18 FGDs across all districts. Each FGD will comprise 7 to 9 participants. In each district, we will also conduct a minimum of 5 IDIs among CHEWs and 4 among clients. This gives a cumulative minimum total of at least 24 IDIs. In addition to the IDIs, we will conduct 5 key informant interviews (KIIs) per district involving health managers such as the district health officer, assistant district health officer in charge of maternal and child health, as well as health facility managers and supervisors. This will give a cumulative minimum total of at least 15 KIIs. An additional 8 KIIs will be conducted at the national level to obtain views from program managers and policymakers. However, data will be collected based on the concept of saturation.

### Sampling Procedure

#### CHEWs and Authorized Cadre: Noninferiority Design

Simple random sampling with replacement will be used to sample CHEWs to observe in each district. In each district, we will construct a sampling frame comprising all trained CHEWs. Simple random sampling (using a Microsoft Excel random number generator) with replacement will be used to identify individual CHEWs that will be observed. The authorized cadre will be selected using a similar approach. Where the number of authorized cadres is smaller than the estimated sample size, we will recruit from neighboring districts because the competence of the authorized cadre should not be dependent on the place where the health worker provides the services.

#### Qualitative Sample

Purposive sampling will be used to identify key stakeholders engaged in family planning activities within the selected districts and at the national level. The respondents will be selected based on their knowledge and experience in offering family planning services. In total, we plan to conduct approximately 65 interviews across the selected intervention districts.

### Study Variables

#### Acceptability of the Community-Based Implant by CHEWs Among the Communities

Acceptability will be measured based on the constructs of the theoretical framework of acceptability [17]. These constructs include (1) affective attitude: how the stakeholders feel about the intervention; (2) burden: the perceived amount of effort that is required to participate in the intervention; (3) ethicality: the extent to which the intervention aligns with an individual’s value system; (4) self-efficacy: the participant’s confidence that they can perform the behaviors required to participate in the intervention; and (5) intervention coherence: the extent to which the participant understands the intervention and how the intervention works.

#### Perceived Effectiveness

Perceived effectiveness is the extent to which the intervention is perceived to be likely to achieve its purpose, and the opportunity costs refer to the extent to which benefits, profits, or values must be given up to engage in the intervention.

#### Perceptions of Community Members Toward the Use of CHEWs to Insert Implants

This is a qualitative variable. We will focus on both positive and negative opinions. We will analyze for convergence and divergence of perceptions.

#### Perceptions of Programmers and Policymakers Toward the Implant Provision Task Shifting to the CHEWs

This is a qualitative variable. We will generate both positive and negative opinions about how the stakeholders perceive the idea of task shifting implant provision to the CHEWs.

#### Competencies of the CHEWs to Insert Implants

We will measure the clinical competencies around implant insertion using an observation checklist. One observation will be made for each CHEW. For each CHEW, we will observe the process from counseling before the implant is inserted until the end, including postinsertion counseling. For each observable item, the CHEW will be marked as having performed the task as satisfactory, unsatisfactory, or not done. Satisfactory will mean that the step or task has been efficiently performed in its proper sequence or individually, unsatisfactory will mean that the task was not performed correctly or in sequence, and not done will mean the task was omitted or not performed, yet it was necessary. Satisfactory will fetch a mark, unsatisfactory will fetch 0.5, and not done=0. CHEWs will be considered competent if they score 80% and above.

### Data Collection Methods

#### Quantitative Data Collection

The quantitative data will be collected through observations among CHEWs and authorized cadres inserting implants as part of the noninferiority assessment. The observation data will be collected using an observation checklist that was developed based on the implant insertion curriculum. The observation data will be used to quantitatively assess competence during the insertion of implants.

#### Qualitative Data Collection

##### Key Informant Interviews

KIIs will be conducted among health workers engaged in the intervention as either trainers or supervisors of the CHEWs, as well as representatives of organizations engaged in provision of long-term reversible family planning. Additionally, those at the policy level, such as district health officers and family planning experts at the Ministry of Health, will be included.

##### Focus Group Discussions

FGDs will be conducted among CHEWs to elicit opinions and perceptions about engaging CHEWs in implant insertion. Additional data about the acceptability of the intervention will also be collected using this approach. The FGDs will comprise 7 to 9 participants. Data will be collected using an FGD guide. Data will be collected in the predominantly spoken local languages, specifically Lango (Lira), Luganda (Kyotera), and Lusoga (Namutumba). All discussions will be audio recorded after obtaining consent.

##### In-Depth Interviews

IDIs will be conducted with CHEWs engaged in the intervention and clients who have had implants inserted by the CHEWs. These interviews will focus on getting individual insights, opinions, and perspectives about the acceptability, feasibility, and quality of services received. They will also generate insights into the experiences during the procedure. The IDIs will be conducted using in-depth guides that have been translated into Langi, Luganda, and Lusoga after obtaining informed consent.

### Data Management

#### Quantitative Data

Quantitative data will be collected on smartphones using electronic questionnaires programmed with Open Data Kit software. Back-to-back encryption will be implemented to protect the respondents’ data. A routine data quality assessment check will be carried out on a daily basis. The IT administrator will check the data received at the server for extreme values, incompleteness, and incorrectly entered fields. A report will be generated based on these findings and immediately communicated to the regional team leaders through the survey coordinator. The final datasets will be uploaded to MakSPH’s secure and password-protected server after cleaning. The data manager and IT administrator will download data from the server and export it to R Software (R Development Core Team) for analysis after necessary cleaning.

#### Qualitative Data

All audio interviews will be transcribed verbatim from the local language to English. In cases where the participants turn down the request to audio record the discussions or interviews, notes will be taken and expanded immediately to enrich the transcripts.

### Data Analysis

#### Quantitative Data Analysis

Quantitative data will be cleaned and analyzed using STATA (version 15), through univariate, bivariate, and multivariate analyses using appropriate statistical tests. For example, the chi-square test for independence will be used to assess whether CHEWs differ from the authorized cadre in the performance of specific tasks involved in the process of inserting implants. The chi-square test is computed based on the null hypothesis that the proportion of CHEWs who perform a specific task is equal to the proportion of the authorized cadre who perform that task. This hypothesis is rejected at a *P* value of .04. The chi-square test statistic is also computed with the assumption that all observations are independent, and therefore, the analysis will control for factors such as the observer, the health facility, and the district that might cause dependence or clustering of observations. Findings will also be presented as proportions, in tables and figures, odds ratios or prevalence ratios, and *P* values at a 95% confidence level.

#### Qualitative Data Analysis

Thematic content analysis techniques will be done using ATLAS.ti software (version 9). The transcripts will be analyzed using a thematic approach following the framework method for the analysis of qualitative data, where data will be compared and contrasted by themes. Thematic network analysis will allow for the generation of relevant themes. Transcripts will first be coded by a multidisciplinary team that includes members with expertise in public health, social sciences, and medicine. Initial codes will be discussed and refined into a working analytic framework during in-person meetings. Later, the initial codes will be applied to all transcripts while allowing new codes to emerge, leading to a final framework that will be agreed upon by the research team. The final framework will be used to develop categories and subsequently themes. The same codebook will be used at the different data analysis time points to allow the team to compare and draw conclusions at the different data collection points.

### Ethical Considerations

#### Overview

Ethical approval was provided by the Makerere University School of Public Health Higher Degrees Research Ethics Committee (approval number SPH-2024‐291). The evaluation protocol was also registered with the Uganda National Council of Science and Technology prior (registration number HS5966ES). We also obtained administrative approval from the respective district local governments before conducting any study procedures.

#### Voluntary Participation

All participants will be informed of the purpose of the study, their right to refuse to participate in the study, and their possible decision not to participate will not be held against them or affect their status (eg, as employees, partners, or beneficiaries). Participants will be enlisted on the basis that they can understand the principles of their involvement in the study, give consent or assent, engage with the study team, and show a willingness to freely express their experiences and opinions.

#### Written Informed Consent

All prospective participants will provide written informed consent before engaging in any study activities. This consent process will involve provision of detailed information on risks, benefits, confidentiality, and the purpose of the research and the voluntary nature of the survey. Participants will be given an opportunity to ask any questions they may have before signing the form.

#### Confidentiality

To ensure privacy and confidentiality, all interviews will be held in private locations. No identifiable data, such as names, ages, addresses, and contact information, will be collected by the research team. All quantitative data will be collected via password-protected tablets that will be transferred to a password-protected server after data collection daily. All final data will be presented only in aggregate to protect confidentiality and safety.

## Results

By August 2025, research assistants who will support the data collection had been trained. Preliminary findings indicated support from facility health workers, which improved confidence and capacity of community health workers to provide implants. Challenges included lack of facilities for waste disposal and lack of tools for record keeping and data management. We anticipate that the data collection will be completed by the end of October 2025, the data analysis will be completed by November 2025, and the final results will be published by December 2026.

## Discussion

### Anticipated Implications

We will discuss the findings and their implications for improving access to family planning services at the community level. We anticipate that in general, the competence of CHEWs in insertion of implants will not differ significantly from that of the authorized cadre. We will compare how task sharing has been used for other service delivery, such as community case finding during integrated management of childhood illnesses, to identify lessons to inform family planning provision. For example, policymakers in Kenya noted concerns about the policy framework within which community health workers can distribute drugs at the community level and how this might affect the delivery of vertically oriented programs [18]. Conversely, task shifting in maternal and child health service delivery has the potential to reduce the workload on authorized cadre to spend more time on tasks that require more advanced training [19].

We will also compare our findings with similar approaches in Burkina Faso [20], Kenya [21], Ethiopia [10], Ghana [22], and Nigeria [11]. In Burkina Faso, health providers, community representatives, government officials, and clients were generally agreeable with the idea of implants being provided by trained community health workers [20]. However, community health workers in Burkina Faso were trained to provide several family planning options in addition to the implants. In Kenya, 95% of 434 respondents agreed that task sharing can increase and expand access to family planning services. It was found to benefit both clients and health service providers, particularly where the community health workers were provided with training, support supervision, and conducted within the requisite policy environment [21]. Similarly, qualitative findings from Ghana revealed that community health nurses could provide safe intrauterine devices to clients if they were well trained and provided with the requisite resources and technical support supervision [22].

Findings will also be compared with other studies that used similar noninferiority designs that assessed for safety, quality, and acceptability of community health workers providing the implants in rural settings [23]. We will also discuss other factors to be considered before scale-up, such as the influence of the fact that the services are provided by CHEWs on cost, method choice, and uptake [24], and the broader impact on health system performance [25].

Methodological strengths and limitations will also be discussed to inform future research.

### Strengths and Limitations

This study has several strengths and limitations. The study applies a noninferior design, which is methodologically more rigorous compared with pre-post designs that have been used for assessment in previous studies [10,11]. In addition, the study uses a convergent parallel mixed methods design, which allows for the collection of both quantitative and qualitative data to reinforce each other. The limitations are the risk of clustering of observations around factors such as the district, the health facility where observations are made, and the observers making the observations. However, we will adjust for clustering in the quantitative analysis.

### Dissemination Plan

Findings will be disseminated to the districts where the study took place, the Ministry of Health, and other stakeholders to inform scale-up. Findings will also be disseminated to the scientific community through abstracts and scientific manuscripts.

## Abbreviations

CHEWs: community health extension workers
FGD: focus group discussion
IDI: in-depth interview
KII: key informant interview
